# Autophagy Induction by Silibinin Positively Contributes to Its Anti-Metastatic Capacity via AMPK/mTOR Pathway in Renal Cell Carcinoma

**DOI:** 10.3390/ijms16048415

**Published:** 2015-04-15

**Authors:** Feng Li, Zhenkun Ma, Zhenfeng Guan, Yule Chen, Kaijie Wu, Peng Guo, Xinyang Wang, Dalin He, Jin Zeng

**Affiliations:** 1 Department of Urology, the First Affiliated Hospital of Xi’an Jiaotong University, Xi’an 710061, China; E-Mails: tctx880315@gmail.com (F.L.); mazhenkun.8@stu.xjtu.edu.cn (Z.M.); guanzhenfeng@163.com (Z.G.); xdwyd@stu.xjtu.edu.cn (Y.C.); yidaiwujin@126.com (K.W.); guopeng661@mail.xjtu.edu.cn (P.G.); 2 Key Laboratory of Environment and Genes Related to Diseases, Ministry of Education of China, Xi’an 710061, China; E-Mail: wangxinyang0929@163.com

**Keywords:** renal cell carcinoma, silibinin, autophagy, AMPK, migration, invasion

## Abstract

Silibinin, a dietary cancer chemopreventive flavonoid from the seeds of milk thistle, has been reported to exhibit anti-metastatic effects on renal cell carcinoma (RCC), but the mechanism underlying this phenomenon is not fully understood. The present study aimed at examining the potential role of autophagy in regulating silibinin-induced anti-metastatic effects on RCC cells. Using RCC ACHN and 786-O cells as a model system *in vitro*, we found that silibinin treatment increased the expression of LC3-II, resulted in the formation of autophagolysosome vacuoles, and caused a punctate fluorescence pattern with the monomeric red fluorescence protein-enhanced green fluorescence protein-LC3 (mRFP-EGFP-LC3) protein, which all are markers for cellular autophagy. Autophagy flux was induced by silibinin in RCC cells, as determined by LC3 turnover assay. Mechanically, the adenosine 5'-monophosphate activated protein kinase (AMPK)/mammalian target of rapamycin (mTOR) pathway was identified as involved in regulation of silibinin-induced autophagy. Furthermore, autophagy induction was demonstrated to positively contribute to silibinin-induced anti-metastatic effects on RCC cells *in vitro*. Activation of autophagy enhanced silibinin-induced inhibition of migration and invasion of RCC cells, while inhibition of autophagy attenuated it. These findings thus provide new information about the potential link between autophagy and metastasis inhibition induced by silibinin, and the induction of autophagy may shed some light into future treatment strategies for metastatic RCC.

## 1. Introduction

In neoplasms deriving from kidney, 90%–95% was renal cell carcinoma (RCC) and it accounted for nearly 3.8% of adult malignancy in 2014 [1]. The incidence of RCC has been increasing globally over the past two decades [2]. Approximately 20%–30% of RCC patients have metastatic disease at first diagnosis, and more than 95% patients have multiple metastases [3]. Patients with stage IV RCC have a significantly reduced five-year survival rate (<30%) [4]. Immunomodulatory therapies using interleukin-2 and/or interferon-α (IFN-α) are recommended for the treatment of metastatic RCC. However, the majority of patients do not benefit from these therapies [5].

Recent advances in understanding the genetics and pathology of RCC have led to many novel target approaches for the treatment of metastatic RCC with higher response rates and lower side effects. Sunitinib, a rationally designed small molecule that inhibits members of receptor tyrosine kinase family, yields clinical benefit in RCC patients, who have longer progression-free survival and overall survival compared to treatment with IFN-α. However, after 6–15 months, disease progression usually occurs [6,7]. Furthermore, it is too expensive in terms of drug costs to be widely used in developing countries. Therefore, novel therapeutic strategies for RCC with high efficacy and low cost are urgently needed.

Silibinin, a major flavonolignan isolated from milk thistle seeds, is being used clinically as a hepatoprotective and antioxidant agent in Asia and Europe. A bulk of evidence from others and us suggests that silibinin exerts significant anti-neoplastic effects in various cancer models both *in vivo* and *in vitro*, including cancers of the skin, breast, lung, colon, bladder, prostate, and kidney [8,9,10,11,12,13,14]. Inhibition of invasion and metastasis has been identified as one of the manifold inhibitory effects of silibinin against cancer. In RCC, silibinin inhibits the invasion and migration of RCC 786-O cells *in vitro*, which is associated with suppressed expression of metalloproteinase (MMP)-2, -9, and urokinase plasminogen activator, and inhibition of mitogen-activated protein kinase (MAPK) pathway signaling [15]. A study conducted by our group has also shown that silibinin inhibits EGFR-induced migration and invasion of RCC cells via a blockade of EGFR/MMP-9 signaling [16]. However, the exact mechanisms responsible for the anti-metastatic effects of silibinin are yet to be elucidated.

Autophagy, an evolutionarily conserved lysosomal self-eating process degrading cytoplasmic proteins and organelles, is responsible for the recycling of metabolic substances and the maintenance of cellular homeostasis [17]. Recent evidence suggests that autophagy may act as a “double-edged sword” with regard to tumor metastasis. Autophagy may inhibit metastasis by promoting anti-tumor inflammatory responses or by inhibiting the expansion of tumor cells into macrometastases. On the other hand, self-eating may promote metastasis by enhancing tumor cell adaptation in response to microenvironmental stresses during metastatic progression [18,19,20]. So there is considerable interest in elucidating the mechanisms of interplay between autophagy and metastasis. Therefore, the purpose of this study is to examine the possible role of autophagy in regulating silibinin-induced anti-metastatic effects on RCC cells. In the present study, we also explored the molecular mechanisms of silibinin-induced autophagy, focusing on adenosine 5'-monophosphate activated protein kinase (AMPK)-regulated autophagic pathway.

## 2. Results

### 2.1. Silibinin Induces Autophagic Vacuoles in Renal Cell Carcinoma (RCC) ACHN and 786-O Cell Lines

**Figure 1:**
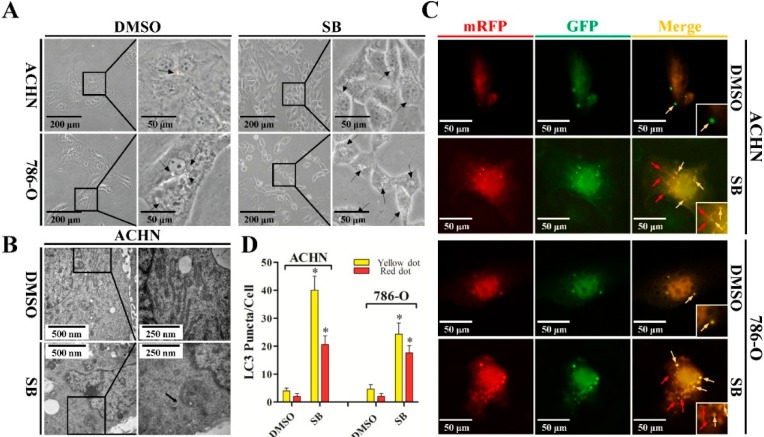
Silibinin induces autophagic vacuoles in RCC ACHN and 786-O cell lines. (**A**) Morphological changes of RCC ACHN and 786-O cells after treatment with 50 μM silibinin (SB) as indicated for 24 h by inverted phase contrast microscopy (×40 and ×100). The regions indicated by rectangles are shown with higher magnification. Arrows point to cytoplasmic vacuole accumulation; (**B**) Representative electron micrographs of cells treated with 50 μM silibinin for 24 h (×20,000 and ×40,000). Arrows point to distinct autophagic structures; (**C**) Examples of cells transiently transfected with ptfLC3 plasmid and treated with 50 μM silibinin for 24 h under fluorescence microscope (×200). Yellow arrows point to autophagosomes and red arrows point to autolysosomes; and (**D**) Quantification of the number of autophagosomes (yellow LC3 puncta) and autolysosomes (red LC3 puncta) per cell. Error bars represent SDs. * *p* < 0.05.

Since there was no report on whether silibinin could induce autophagy-like morphological change in RCC cells, we firstly treated ACHN and 786-O cell lines with 50 μM of silibinin and investigated the morphological change under an inverted microscope. As shown in Figure 1A, RCC cells treated with silibinin showed morphological features of cytoplasmic vacuole accumulation. To examine whether cell vacuolation induced by silibinin was related to autophagy, the ultrastructure of the cells was analyzed by transmission electron microscopy. A significant increase in the autophagic double-membrane compartments containing lamellar structures in silibinin-treated cells was observed (Figure 1B).

A hallmark of mammalian autophagy is the conversion of LC3-I to LC3-II via proteolytic cleavage and lipidation, which is then covalently modified and localized to autophagic vacuoles during autophagy induction. We next transiently transfected a pH-sensitive LC3 construct consisting of a tandem fusion of the acid-insensitive mRFP and the acid-sensitive EGFP into RCC cells to address the effects of silibinin on autophagosome formation. The mRFP-EGFP-LC3 reporter plasmid (ptfLC3) will emit yellow (green merged with red) fluorescence in non-acidic structures (including autophagosomes), but appears as red only in autolysosomes due to the quenching of GFP in these structures with low pH. As shown in Figure 1C,D, a pronounced increase in both yellow and red puncta was also observed in RCC cells treated with silibinin. These data suggest that silibinin treatment enhances autophagosome numbers and autophagosome maturation in RCC cells.

### 2.2. Silibinin Induces Autophagic Flux in RCC ACHN and 786-O Cells

**Figure 2:**
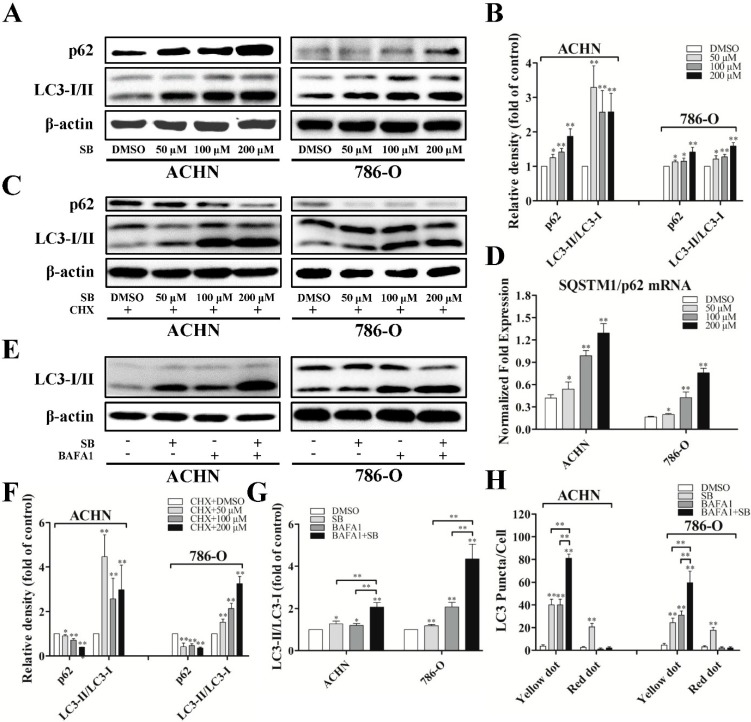
Silibinin induces autophagic flux in RCC ACHN and 786-O cells. (**A**) Cells were treated with indicated dose of silibinin (SB) for 24 h, the levels of p62 and LC3-I/II were checked by Western blot and quantitative analyses were done (**B**); (**C**,**F**) RCC cells were treated with the indicated dose of silibinin for 24 h in the presence and absence of 350 nM cyclohexamide and the levels of p62 and LC3-I/II were examined; (**D**) The expression of SQSTM1/p62 mRNA accessed by real-time PCR. RCC cells were treated with 50 μM of silibinin for 24 h in the presence and absence of 10 nM BAFA1 and the levels of LC3-I/II (**E**,**G**) and the numbers of LC3 puncta (**H**) were examined. Blots are representative of three separate experiments. Error bars represent SDs. * *p* < 0.05; ** *p* < 0.01.

Since we have observed the re-distribution of LC3-II to autophagosomes and autolysosomes, we further examined the expression of LC3-I/II using western blot. Overnight treatment of RCC ACHN and 786-O cells with silibinin resulted in a significant increase of LC3-II accumulation compared with vehicle (DMSO)-treated cells, which followed a dose-response trend (Figure 2A,B). More importantly, silibinin could increase the level of LC3-II at very low doses and at earlier times which did not have any impacts on cells’ viability (Figure S1).

To examine autophagic clearance, the levels of SQSTM1/p62, a classical macroautophagy substrate, were then investigated. This revealed a surprising increase in total levels of p62 upon treatment with silibinin (Figure 2A,B). Additionally, the mRNA level of p62 was also upregulated, as determined by real-time PCR (Figure 2D). Based upon the upregulation of p62 in both mRNA and protein level, silibinin treatment was carried out in the presence of cyclohexamide, an inhibitor of protein synthesis. As shown in Figure 2C,F, the silibinin-mediated increase in p62 levels could be blocked by cyclohexamide and silibinin treatment resulted in a significant decrease of p62 level in the presence of cyclohexamide, suggesting the induction of autophagic clearance by silibinin.

To further confirm the induction of autophagic flux by silibinin, the response of RCC cells to silibinin was assessed in the presence of bafilomycin A1 (BAFA1), an inhibitor of lysosomal acidification. As expected, LC3-II levels and LC3 yellow puncta were increased in both DMSO- and silibinin-treated cells in the presence of BAFA1, with the silibinin plus BAFA1 treated cells displaying a significantly higher level of LC3-II and yellow puncta compared to cells treated with silibinin alone or BAFA1 alone (Figure 2E,G,H). This suggests that the increase in LC3-II levels by silibinin is due to an increase in production rather than the decreased recycling of LC3-II.

### 2.3. Silibinin Induces Autophagy through AMPK/Mammalian Target of Rapamycin (mTOR) Pathway

To further understand the underlying mechanism of autophagy induced by silibinin, we examined the expression of ATG genes related to autophagy. However, no significant changes were observed after silibinin treatment at both mRNA and protein levels (Figure S2).

**Figure 3:**
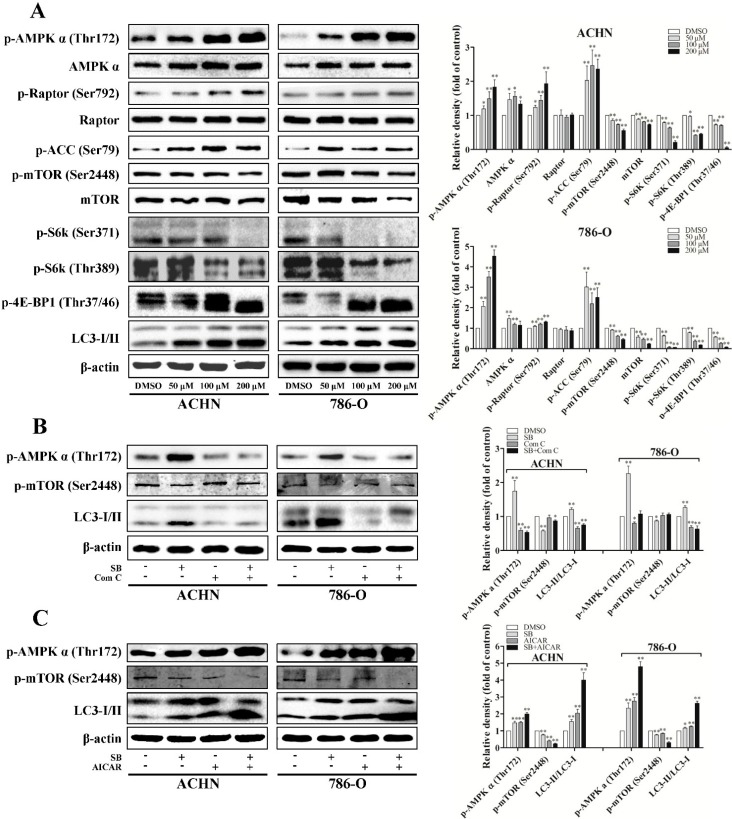
Silibinin induces autophagy through the AMPK/mTOR pathway. (**A**) Cells were treated with indicated doses of silibinin (SB) for 24 h and the protein levels of p-AMPKα, p-ACC, p-Raptor, p-mTOR, p-S6K, and p-4E-BP1 were checked. RCC cells were pre-incubated with AMPK inhibitor Compound C (Com C, 5 μM) (**B**) or activator AICAR (1 mM) (**C**) for 4 h and then treated with silibinin (50 μM) for 24 h. The levels of p-AMPKα, p-mTOR, and LC3-I/II were examined. Blots are representative of three separate experiments. Error bars represent SDs. * *p* < 0.05; ** *p* < 0.01.

mTOR complex 1 (mTORC1) signaling is critical for autophagy induction, with activated mTORC1 suppressing autophagy, and negative regulation of mTORC1 inducing it. mTORC1 regulates protein synthesis through the phosphorylation and inactivation of the repressor of mRNA translation, eukaryotic initiation factor 4E-binding protein (4E-BP1), and through the phosphorylation and activation of S6 kinase (S6K). To examine the role of mTORC1 signaling in silibinin-induced autophagy, we determined the phosphorylation levels of mTOR, 4E-BP1, and S6K. Our results showed that silibinin inhibited the phosphorylation levels of mTOR at Ser-2448, 4E-BP1 at Thr-37/46, and S6K at Thr-389 and Ser-371 (Figure 3A).

Furthermore, autophagy can also be induced by AMP-activated protein kinase (AMPK), which is a key energy sensor and regulates cellular homeostasis. As recent studies imply the cross-talking between AMPK and mTORC1 signaling in regulating autophagy, we evaluated the effects of silibinin on AMPK activity. Our results showed that silibinin treatment significantly increased the level of phospho-ACC (Ser79) and phospho-AMPK (Thr172), suggesting the activation of AMPK signaling (Figure 3A–C). The regulatory associated protein of mTOR (Raptor) is an mTOR binding partner that mediates mTOR signaling to downstream substrates. Phosphorylation of Raptor by AMPK at Ser722/Ser792 has been demonstrated to be essential for inhibition of the mTORC1 signaling. Consistent with the significant inhibition of mTORC1 activity, we also observed the increased phosphorylation of Raptor at Ser792 after silibinin treatment (Figure 3A).

To confirm that the increase of AMPK phosphorylation was involved in the inhibition of mTOR during autophagy, we pre-inhibited AMPK by compound C or pre-activated it by AICAR before silibinin was added. As expected, the effects of silibinin on phospho-mTOR and LC3-II can be reversed by compound C (Figure 3B) and enhanced by AICAR (Figure 3C).

Collectively, these findings suggest that the AMPK/mTOR pathway is involved in the induction of autophagy by silibinin in RCC cells.

### 2.4. Autophagy Induced by Silibinin Contributes to Its Anti-Metastatic Effects

**Figure 4:**
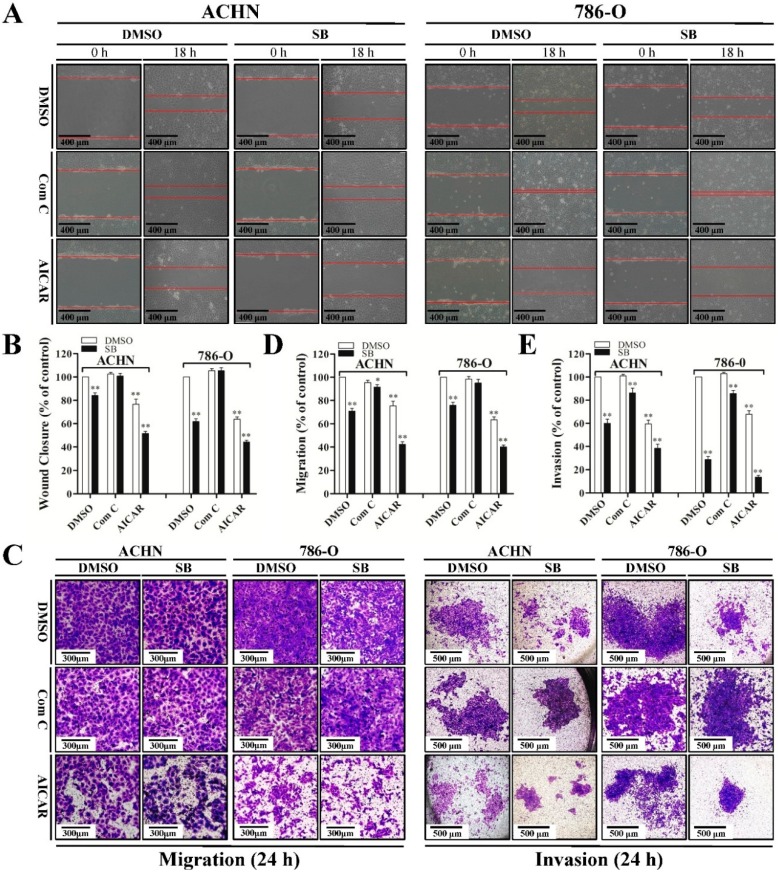
Autophagy induced by silibinin contributes to its anti-metastatic effects. Cells were pre-treated with compound C (Com C, 5 μM) or AICAR (1 mM) for 4 h and treated with silibinin (SB, 50 μM) for the indicated time (18 or 24 h). Wound healing assay (**A**,**B**) and trans-well migration (**C**,**D**) and invasion (**C**,**E**) assay were performed. Red lines represent the borders of the wounds. Pictures shown are representative of three separate experiments. Error bars represent SDs. * *p* < 0.05; ** *p* < 0.01.

Since siliblilin could induce both autophagy and inhibition of cell metastasis in RCC cells, we next investigated the relationship between silibinin-induced autophagy and anti-metastatic properties. When autophagy was inhibited by compound C in RCC cells, silibinin-induced anti-metastatic effects were attenuated, as determined by wound healing assay (Figure 4A,B) and trans-well migration/invasion assay (Figure 4C–E). Conversely, activation of autophagy by AICAR significantly enhanced silibinin-induced anti-metastasis capacity. These findings suggest the interplay between silibinin-induced autophagy and anti-metastatic effects in RCC cells.

## 3. Discussion

Silibinin has been proven to have anti-cancer capacity in RCC both *in vitro* and *in vivo*. Its anti-cancer effects are associated with proliferation inhibition, apoptosis induction, and anti-metastasis [13,15,16]. In the present study, we first documented that silibinin induced early autophagy, which subsequently contributed to inhibition of migration and invasion in RCC ACHN and 786-O cells *in vitro*, involving activation of AMPK/mTOR pathway.

Induction of autophagy by anticancer reagents has been widely studied in a number of cancer cell models. Initially, autophagy was regarded as a specific type of cell death, since it is essentially a self-cannibalizing event. However, an increasing body of evidence is pointing to the idea that autophagy may play dual roles in carcinogenesis and anticancer therapies [21,22,23]. When cells are subjected to adverse stress, such as nutrient starvation or chemotherapeutic agents, cancer cells may trigger the autophagic response that degrades unnecessary molecules or organelles to promote cellular survival. On the other hand, treatment with many anticancer reagents or ionizing radiation has been shown to induce autophagic cell death, directly leading to inhibition of cancer [21,22,23]. Silibinin has been reported to induce autophagy in fibrosarcoma and colorectal cancer [24,25,26]. In our study, we first demonstrate that silibinin induced autophagy in RCC cells, as determined by electron microscopy, LC3 turnover assay, and tandem fluorescence microscopy. Interestingly, the level of p62 was increased, although degradation of p62 after silibinin treatment was observed when pretreated with cyclohexamide. Further research is needed to clarify the mechanisms of both transcriptional and translational upregulation of p62 by silibinin.

Several critical molecules and pathways have been shown to regulate autophagy; of these, mTOR and AMPK pathway have been best characterized, and received the most attention from researchers [27]. Our further mechanistic studies revealed that the effects of silibinin on autophagy induction were found to be mTOR-dependent and regulated by AMPK. Silibinin strongly inhibited the activation of the mTOR pathway but activated the AMPK pathway. However, we failed to observe the activation of Beclin-1, which played a vital role in the initiation of autophagosomes [28]. Recently, a couple of studies revealed that oxidative stress and calcium signaling can also regulate mTOR signaling pathway by activating AMPK [29,30]. Based on the significant regulation of AMPK/mTOR pathway by silibinin, there is considerable interest in elucidating the effects of silibinin on reactive oxygen species (ROS) and calcium signaling. Initially, the hepatoprotective agent silibinin is well known for its strong antioxidant properties, including scavenging of ROS [31,32]. A recent study showed that silibinin rapidly induced oxidative stress in colorectal cancer SW480 cells due to ROS generation with a concomitant dissipation of mitochondrial potential and cytochrome c release leading to mild apoptosis. Interestingly, with increased exposure to silibinin, further release of cytochrome c and apoptotic response were inhibited, which was correlated with increased autophagy [33]. However, the relationship between ROS generation and autophagy induction by silibinin remains unclear. Similarly, silibinin was reported to induce a transient increase in intracellular Ca^2+^ followed by an increase in ROS generation in glioma cells [34]. However, a functional link between autophagy and calcium signaling or ROS generation has not been illustrated by this study. We also observed that silibinin could induce autophagy at the low dose of 20 μM and at as early as eight hours at low dose (50 μM), which did not affect cells’ viability. Evidence from others and us has demonstrated that silibinin could inhibit the migration and invasion of RCC cells at the low dose of 50 μM [13,16]. Additionally, autophagy is considered to serve both pro- and anti-metastatic functions depending on the contextual demands placed on cancer cells throughout the metastatic process. The relationship between autophagy and cancer metastasis is still controversial [19,20]. Thus it would be of considerable interest to identify the potential relationship between silibinin-induced autophagy and metastasis inhibition. In our study, inhibition of autophagy by compound C (AMPK inhibitor) or activation of autophagy by AICAR (AMPK activator) significantly attenuated or enhanced the anti-metastatic effects of silibinin, suggesting the anti-metastatic functions of autophagy induced by silibinin. It is still unclear how autophagy induction contributes to metastasis inhibition. It was reported that death effector domain-containing DNA-binding protein (DEDD), a key effector molecule for cell death signaling receptors, physically interacted with the class III PtdIns 3-kinase complex containing, which controlled the initiation steps of autophagy. This interaction activated autophagy and induced the autophagy-mediated lysosomal degradation of Snail and Twist, two master inducers of the metastatic process [35,36]. One recent study showed that autophagy might act as a suppressor of metastasis by preventing p62-dependent stabilization of the metastasis-promoting transcription factor Twist1 [37]. However, further studies are needed to illustrate the molecular mechanisms governing the interplay between autophagy and metastasis inhibition.

Our study focused on the relationship between autophagy induction and metastasis inhibition in RCC cells. Accumulating evidence has demonstrated the involvement of autophagy in tyrosine kinase inhibitors (TKIs)-based targeted therapy in RCC, although its exact role is still unclear. It was reported that autophagy inhibition enhanced sorafenib activity, causing substantial cell apoptosis in RCC cells [38]. Conversely, sunitinib treatment triggered incomplete autophagy and stimulated a lysosomal-dependent necrosis, while pazopanib treatment induced autophagic cell death in bladder cancer cells [39], suggesting the positive contribution of autophagy to cancer cell death induced by TKIs. Additionally, stimulation of autophagy was demonstrated to be responsible for one of the mechanisms underlying resistance to everolimus, an inhibitor of mTORC1 [40]. Since autophagy has been considered to act as a “double-edged sword” with regard to cancer progression, different responses of cancer cells to autophagy induction or inhibition may be observed in different model systems. Both TKIs and everolimus have been approved in RCC patients; however, their efficacy is limited by feedback loops and cross-talking with other signaling pathways, leading to the development of drug resistance [41]. Since silibinin is a nontoxic natural agent with multiple targets, common TKIs or mTOR-based targeted therapies combined with silibinin may exert enhanced therapeutic effects through synergistic response or compensation of inverse properties. Combination treatment may also decrease the systemic toxicity caused by targeted therapy, because lower doses might be used. However, what are the effects of the combination of targeted drugs and silibinin on RCC? Are there any interactions between them? The answers to these questions clearly require more focus and further research.

## 4. Experimental Section

### 4.1. Cell Culture

RCC cell lines 786-O and ACHN cell lines were purchased from American Type Culture Collection (Manassas, VA, USA). ACHN and 786-O cell lines were cultured in Eagle’s Minimum Essential Medium (EMEM) and RMPI-1640 medium, respectively. Culture medium was supplemented with 10% fetal bovine serum and 100 U/mL penicillin and 0.1 mg/mL streptomycin (Gibco, Grand Island, NY, USA). Cells were incubated in a humidified atmosphere containing 5% CO_2_ at 37 °C and observed by inverted microscope (Olympus, Tokyo, Japan) (×100 and ×200).

### 4.2. Regents, Antibodies, and Plasmid

Silibinin was purchased from Sigma–Aldrich (St. Louis, MO, USA). Bafilomycin A1 (BAFA1) was purchased from EMD Millipore (Darmstadt, Germany). Compound C and AICAR were purchased from Abcam (Cambridge, UK). Lipofectamine 2000 reagent was purchased from Invitrogen (Carlsbad, CA, USA). RIPA buffer was purchased from Cell Signaling Technology (Boston, MA, USA), protease inhibitor and phosphatase inhibitor were from Roche (Basel, Switzerland). BCA qualification system was purchased from Pierce (Rockford, IL, USA). Primary antibodies against LC3-I/II, ATG3, ATG5, ATG7, p-Raptor (Ser792), Raptor, mTOR, p-mTOR (Ser2448), p-ACC (Ser79), p-AMPK α (Thr172), AMPK α, p-S6K (Ser371), p-S6K (Thr389), p-4E-BP1 (Thr37/46), β-actin, and peroxidase-conjugated secondary antibodies were purchased from Cell Signaling Technology (Boston, MA, USA), p62 was from Novus (Littleton, CO, USA). PVDF membrane was purchased from Bio-rad (Hercules, CA, USA). The mRFP-EGFP-LC3 reporter plasmid (ptfLC3) was a gift from Tamotsu Yoshimori (Addgene plasmid # 21074) [42].

### 4.3. Tandem Fluorescence Microscopy

Cells were seeded onto coverslip and transient transfected with tandem fluorescent ptfLC3 expressing plasmid with Lipofectamine 2000 reagent. After 24 h transfection, cells were treated with silibinin for an additional 24 h. Cells were then fixed by 4% paraformaldehyde. The localization of LC3 puncta was observed by fluorescence microscopy (×200, Olympus, Tokyo, Japan).

### 4.4. Transmission Electron Microscopy

Cells were treated with silibinin for 24 h and fixed in Karnovsky’s fixative (2% paraformaldehyde and 5% glutaraldehyde in 0.1 M cacodylate, pH 7.4) followed by osmium tetroxide. Samples were then dehydrated in ethanol, infiltrated and embedded with TAAB Low Viscosity Resin (TLV) mixture at 60 °C for 24 h, and sectioned to 80 nm in thickness on 300 mesh copper slot grids. Analysis was performed by transmission electron microscopy (JEOL, JEM-1400, Tokyo, Japan) (×20,000 and ×40,000).

### 4.5. Western Blot

Cells were washed with ice-cold phosphate-buffered saline (PBS) and then solubilized in RIPA buffer containing protease inhibitor and phosphatase inhibitor. Lysates were qualified by BCA qualification system. Then the protein was loaded onto 12% SDS-PAGE and transferred to a polyvinylidene fluoride (PVDF) membrane. Membranes were immune-blotted with indicated primary antibodies overnight at 4 °C followed by peroxidase-conjugated secondary antibody for 1 h at room temperature. The bands were visualized by ECL system (Bio-Rad, Hercules, CA, USA). Band densities were measured and analyzed.

### 4.6. Wound Healing Assay

Cells were seeded onto six-well plates. Wounds were scratched when cells had grown to 100% confluence by 200-μL pipet tips. The culture medium was exchanged for a fresh medium containing silibinin (50 μM) for 24 h, with or without AICAR (1 mM) or compound C (5 μM) pretreatment. Wound closure was monitored at designated time points by inverted microscope (×50).

### 4.7. Trans-Well Migration and Invasion Assay

Cells were seeded onto six-well plates and grew to 60% confluence. Then cells were treated with silibinin (50 μM) for 24 h, with or without AICAR (1 mM) or compound C (5 μM) pretreatment. Cells were digested and centrifuged at 1000 rpm for 5 min. Twenty-five thousand cells were seeded onto a mini-cell (EMD Millipore, Darmstadt, Germany) without matrix gel (migration assay) and 80,000 cells were seeded onto a mini-cell with matrix gel (invasion assay) in 200 μL serum-free medium. The lower chamber was filled with 800 μL medium with 10% FBS. After incubation for 24 h at 37 °C in 5% CO_2_, cells were fixed with 4% paraformaldehyde and stained by crystal violet. Cell numbers were counted in three random fields (×100) per mini-cell.

### 4.8. Quantitative Real-Time Polymerase Chain Reaction (PCR) Analysis

Total RNA from cells was extracted using fasten 2000 RNA extract kit (Shanghai, China) following the manufacturer’s protocol. Reverse transcription was performed with 2 μg RNA using Takara reverse kit (Dalian, China). SYBR green reaction mix (Takara) was used to perform RT-PCR following the manufacturer’s instructions. Primer sequences used are shown in supplemental Table S1. The experiment was performed in four individual trials in triplicate. β2 mircoglobulin (β2MG) was used as the control.

### 4.9. Statistical Analysis

All experiments were performed at least three times. The results were presented as the mean ± standard deviation (SD). One-way ANOVA was performed to analyze differences between experimental groups. Image analyzer Image J (NIH, Bethesda, MD, USA) was used to quantify LC3 puncta numbers, wound closure, and migrated and invaded cells, and to measure the densities of bands in Western Blot. A *p* value <0.05 was considered statistically significant in all cases.

## 5. Conclusions

Taken together, silibinin induces autophagy via the AMPK/mTOR pathway in RCC cells. Autophagy induction positively contributes to silibinin-induced inhibition of migration and invasion of RCC cells *in vitro*. These findings provide new information about the potential link between autophagy and metastasis inhibition induced by silibinin, and the induction of autophagy may shed some light into future treatment strategies for metastatic RCC.

## Supplementary Materials

Supplementary materials can be found at http://www.mdpi.com/1422-0067/16/04/8415/s1.
