# Supplementary material for: Autophagy Induction by Silibinin Positively Contributes to Its Anti-Metastatic Capacity via AMPK/mTOR Pathway in Renal Cell Carcinoma
Source: Int J Mol Sci. 2015 Apr 15;16(4):8415–29. doi: 10.3390/ijms16048415 (PMC4425089; doi:10.3390/ijms16048415)
Supplement: Supplementary file 1 [file ijms-16-08415-s001.pdf]

Supplementary Information

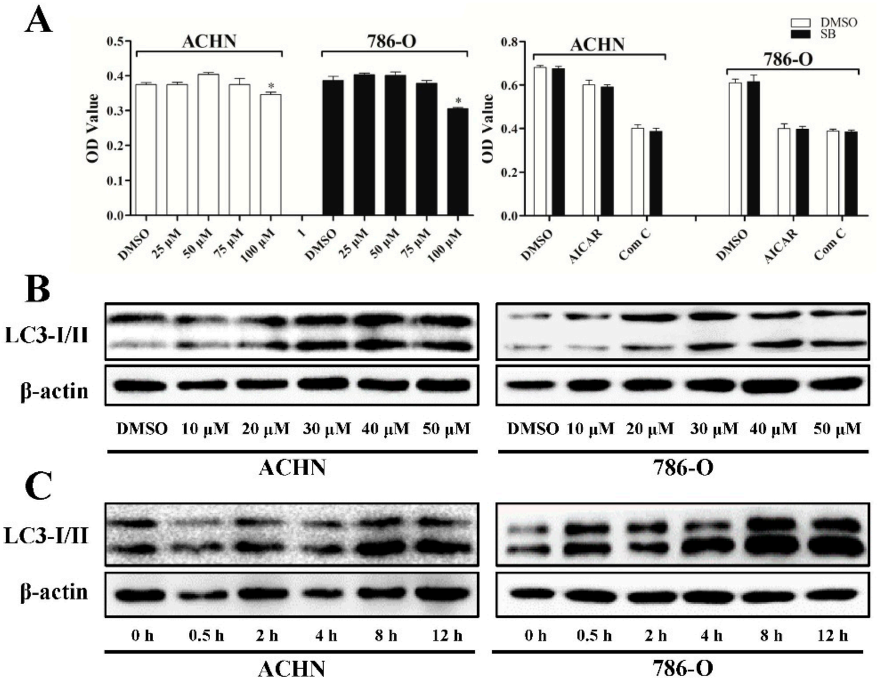

Figure S1. Effects of different doses of silibinin on cell viability and LC3-I/II levels. (**A**) MTT was performed to assess the impact of different doses of silibinin on cells viability after 24 h incubation with or without AICAR (1 mM) or Compound C (5 μM) pretreatment. Cells were treated with (**B**) different doses of silibinin (10, 20, 30, 40, and 50 μM) for 24 h or (**C**) treated with 50 μM of silibinin for different time periods (0, 2, 4, 8, and 12 h); autophagy marker LC3-I/II was checked. β-Actin was used as the loading control. Blots are representative of three separate experiments. Error bars represent SDs. * $p < 0.05$.

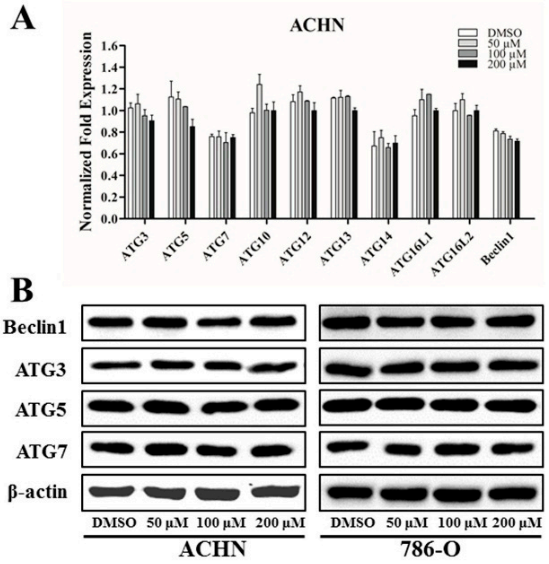

Figure S2. Effects of silibinin on autophagy-related gene expressions. After treatment with silibinin (50, 100, and 200 μM) for 24 h, the expression of autophagy-related ATGs was checked by real-time PCR (**A**) and Western blot (**B**). β2MG and β-actin were used as the control, respectively. Real-time PCR and blots are representative of three separate experiments. Error bars represent SDs.

Table S1. The primers used in real-time PCR.

Gene	Forward	Reverse
β2MG	GAGGCTATCCAGCGTACTCCA	CGGCAGGCATACTCATCTTTT
SQSTM1/p62	GCACCCCAATGTGATCTGC	CGCTACACAAGTCGTAGTCTGG
Beclin1	CCATGCAGGTGAGCTTCGT	GAATCTGCGAGAGACACCATC
ATG3	GACCCCGGTCCTCAAGGAA	TGTAGCCCATTGCCATGTTGG
ATG5	AAAGATGTGCTTCGAGATGTGT	CACTTTGTCAGTTACCAACGTCA
ATG7	CAGTTTGCCCCTTTTAGTAGTGC	CCAGCCGATACTCGTTCAGC
ATG10	AGACCATCAAAGGACTGTTCTGA	GGGTAGATGCTCCTAGATGTGAC
ATG12	CTGCTGGCGACACCAAGAAA	CGTGTTCGCTCTACTGCCC
ATG13	TTGCTATAACTAGGGTGACACCA	CCCAACACGAACTGTCTGGA
ATG14	GCGCCAAATGCGTTCAGAG	AGTCGGCTTAACCTTTCCTTCT
ATG16L1	AACGCTGTGCAGTTCAGTCC	AGCTGCTAAGAGGTAAGATCCA
ATG16L2	TGGACAAGTTCTCAAAGAAGCTG	CCTCAGTGCGACCAGTGAT
